# Psychometric Properties of the Science Self-Efficacy Scale for STEMM Undergraduates

**DOI:** 10.3390/ejihpe15070124

**Published:** 2025-07-04

**Authors:** Jayashri Srinivasan, Krystle P. Cobian, Minjeong Jeon

**Affiliations:** 1 School of Education and Information Studies, University of California, Los Angeles, CA 90095, USA; 2 Geffen School of Medicine, University of California, Los Angeles, CA 90095, USA

**Keywords:** validation study, biomedical research workforce, stem education, scientific self-efficacy, item response theory

## Abstract

Biomedical research training initiatives need rigorous evaluation to achieve national goals of supporting a robust workforce in the biomedical sciences. Higher science self-efficacy is associated with the likelihood of pursuing a science-related research career, but we know little about the psychometric properties of this construct. In this study, we report on a comprehensive validation study of the Science Self-Efficacy Scale using a robust sample of 10,029 undergraduates enrolled across 11 higher education institutions that were part of a biomedical training initiative funded by the National Institutes of Health in the United States. We found the scale to be unidimensional with an Omega hierarchical (ω_h_) reliability coefficient of 0.86 and a marginal reliability of 0.91. Within the item response theory framework, we did not detect variation in item parameters across undergraduates’ race/ethnicity; however, one item had parameters that varied across gender identity. We determined that the Science Self-Efficacy Scale can be employed across undergraduates enrolled in science, and researchers can use the scale across a diverse group of students. Implications include ensuring that the scale functions consistently across diverse populations, enhancing the validity of conclusions that can be drawn from survey data analysis. Validating this construct with item response theory models strengthens its use for future research.

## 1. Introduction

The ongoing need to ensure nations have a robust scientific workforce that supports scientific progress in all sectors of society, including health care, technology, and engineering, is often faced with the challenge of supporting and retaining scientific trainees (Milojević et al., 2018; Dunn & Bourne, 2017; Science and Technology Action Committee [STAC], 2023). Many scientific workforce positions require years of specialized training to develop content expertise and cultivate the capacity to use the scientific method to advance societal knowledge that can address global challenges. Consequently, retaining individuals on a science career pathway is critical for maximizing their dedication to obtaining the highly specialized research skills and expertise needed to address global challenges via scientific research (Nearing et al., 2015; National Academies of Sciences, Engineering, and Medicine [NASEM], 2016, 2024; Science and Technology Action Committee [STAC], 2023).

To effectively study the mechanisms and outcomes of biomedical research training and workforce development initiatives, it is critical to have valid and reliable measures to effectively capture science training efforts. One psychosocial factor known to predict a greater likelihood of pursuing a science-related career is self-efficacy, the personal judgment of how well or poorly individuals can cope with a given challenge based on their skills and the circumstances of the challenge (Bandura, 1977, 1997; Byars & Hackett, 1998; Lent et al., 2000). Self-efficacy theory explains how people think, motivate themselves, and ultimately how they behave, including how long they will persist in the face of obstacles or challenging situations (Bandura & Adams, 1977). Ithas been applied extensively in science, technology, engineering, mathematics, and medicine (STEMM) training program evaluation to both predict and assess the outcomes of training interventions. Research suggests that high levels of self-efficacy predict persistence and achievement (Artino, 2012; Schunk & Mullen, 2012), that self-efficacy beliefs for students from historically underrepresented groups can be improved by college training programs (MacPhee et al., 2013), and that science self-efficacy mediates the relationship between gender and science identity for heterosexual college students (Miles & Naumann, 2021).

Additionally, self-efficacy is domain-specific, meaning that an individual can have high self-efficacy in scientific research skills and lower self-efficacy in nonscientific arenas. Thus, while several self-efficacy constructs have been developed and validated over the past few decades, there is a need for valid and reliable self-efficacy in the domain of science. While several studies have examined self-efficacy beliefs in science (Adedokun et al., 2013; Dorfman & Fortus, 2019; Larose et al., 2006), we focus on the domain of scientific research skills because these skills are broadly critical to many jobs within the scientific workforce regardless of scientific discipline. Thus, we conceptualize science (sometimes also called “research” or “scientific” in the field) self-efficacy as a measure of an individual’s self-competence with respect to scientific skills (Chemers et al., 2011; Estrada et al., 2011; Robnett et al., 2015). 

Chemers et al. (2011) examined the literature on effective practices for supporting the training and retention of undergraduate science trainees, focusing primarily on the role of undergraduate research experiences. With undergraduate research as the focus, they hypothesized that self-efficacy with respect to scientific skills would predict successful academic and professional scientific career outcomes given prior research on self-efficacy and social cognitive career theory (Lent et al., 2000; Lent & Brown, 2002). Studies have found that science self-efficacy corresponds with a higher likelihood of pursuing a research-focused career for both undergraduate and graduate/doctoral students (Adedokun et al., 2013; Chemers et al., 2011; Estrada et al., 2011). Additionally, studies have examined the relationship between science self-efficacy and other psychosocial measures such as science identity (Chemers, 2006) to obtain a more complete understanding of students’ scientific training trajectories (Estrada et al., 2011).

Validation studies that conduct a thorough examination of the psychometric properties of a scale are important because they ensure that the scale measures the target concept and appropriately represents the characteristics of its intended audience (AERA et al., 2014). Clarity on whether the scale used in a study has well-established psychometric properties and whether the scale operates similarly in different subgroups of a population ultimately enhances the trustworthiness of the results and implications of studies that employ validated scales.

The current study examines the psychometric properties of the Science Self-Efficacy Scale (SSES), initially developed by Chemers et al. (2011) with 10 items and adapted into a six-item scale by Estrada et al. (2011). This six-item scale was used in the Enhance Diversity Study (EDS), a program assessment examining biomedical research capacity-building initiatives at 11 higher education institutions across the United States. At the time of the EDS, the adapted six-item SSES had not yet been validated using a large, national sample of diverse undergraduate students.

We conducted a comprehensive validation of the scale by examining (a) construct validity, which includes an analysis of reliability coefficients, fitting an item response theory model, and assessing measurement invariance of how the scale functions across various subgroups, such as gender and race/ethnicity; (b) dimensionality using a cross-validation approach; and (c) validity based on other related traits or outcomes. By employing an item response theory framework, we were able to examine the specific item characteristics of the SSES and its relationship to the science self-efficacy latent construct. The results of this validation study provide critical information for researchers, evaluators, faculty, and administrators involved in initiatives that aim to facilitate retention in scientific career training environments.

## 2. Methods

### 2.1. Data Source

The current study analyzed data from the EDS (Guerrero et al., 2022), implemented by the Coordination and Evaluation Center (CEC) of the National Institutes of Health (NIH) Diversity Program Consortium at the University of California, Los Angeles. The EDS was part of the larger BUilding Infrastructure Leading to Diversity (BUILD) initiative funded by the NIH to implement and study various approaches to engage and retain students from diverse backgrounds in the biomedical research workforce. As a national multi-site longitudinal study, BUILD aimed to broaden participation across scientific professional fields. EDS survey data were collected across 11 higher education institutions. The survey employed for these analyses was the Student Annual Follow-up Survey (SAFS), which was developed and administered by the CEC every spring from 2016 to 2022. The SAFS collected students’ perceptions and views on various educational and career goals, as well as their experiences in college. For the current study, we used the SAFS 2019 data set because the survey was conducted before the global disruptions of the COVID-19 pandemic, which likely impacted students’ responses to the items in the following years. Furthermore, data collection became more consistent by 2019 in terms of compensation and communication to potential student participants (Ramirez et al., 2022). Additionally, we included institutional records data for undergraduates, which provided us with information on students who graduated by 2022.

### 2.2. Science Self-Efficacy Scale (SSES)

As noted earlier, the SSES is a six-item scale. Items include the following: (1) use technical science skills (use of tools, instruments, and/or techniques), (2) generate a research question, (3) determine how to collect appropriate data, (4) explain the results of a study, (5) use scientific literature to guide research, and (6) integrate results from multiple studies. Responses are recorded on a 5-point Likert scale ranging from “not confident at all” to “absolutely confident.” The last option is “I choose not to answer,” which was set to missing data for our analyses.

### 2.3. Analyses

#### 2.3.1. Descriptive Analysis

To examine the psychometric properties of the construct, we first conducted descriptive analysis, including examining the demographic characteristics of the sample, the response percentages, and the inter-item correlations among the items.

In terms of handling missing data, we found approximately 8% of missing responses to the six items. In order to conduct a factor analysis across the items and to fit an item response theory model to the six items, it is vital to have complete cases. To this end, we conducted a listwise deletion of students who had missing responses (Not Applicable, or N/A) across all six items; that is, if a student had N/A for all six items, these cases were dropped from the analysis. For gender and race/ethnicity, we also conducted listwise deletion. We did not conduct any tests to examine missing data mechanisms because it would not be appropriate to impute gender and race/ethnicity for students.

#### 2.3.2. Construct Validity: Dimensionality, Reliability Coefficients, and Item Response Theory Modeling

To assess the construct validity of the scale, we examined the dimensionality of the construct. We conducted the Kaiser–Meyer–Olkin (KMO) test to determine the suitability of data for factor analysis. Next, we split the data set into two halves and used a cross-validation approach to assess the dimensionality (Floyd & Widaman, 1995). With the first half of the data set, we conducted an exploratory factor analysis (EFA) with a varimax rotation and used the second half of the data set to cross-validate whether the factor structure was held by conducting a confirmatory factor analysis (CFA). To assess the model fit statistics, we used the standardized root mean square residual (SRMSR), the Tucker–Lewis Index (TLI), and the root mean square error of approximation (RMSEA) index. The TLI is expected to be greater than 0.90 and the RMSEA should be less than 0.05, indicating good model fit (Hu & Bentler, 1999). We conducted the analyses using R software (R Core Team, 2022). It is important to note that the data set used for the current study is large and encompasses diverse demographic characteristics (see Table 1). The common rule of thumb for assessing construct validity and reliability is that there should be at least 10 participants for each item of the scale, and the ideal scenario is to have at least 15 or 20 participants for each item (Clark & Watson, 1995).

Second, after establishing unidimensionality, we examined three measures for the reliability coefficients. Historically, the Cronbach’s alpha has been the most popular statistic for assessing reliability; however, it assumes normality, which posed a threat to our interpretations given that we had 5-point Likert scale items. In recent years, multiple researchers have indicated the need to move away from the Cronbach’s alpha when there are multiple items comprising a construct or when there are multi-dimensional scales (Green & Yang, 2009; McDonald, 2013; Trizano-Hermosilla & Alvarado, 2016; Zinbarg et al., 2005). In general, while the values of the reliability coefficients range between 0 and 1, an acceptable value is greater than 0.70 (Cronbach, 1951; McNeish, 2018). In addition to the Cronbach’s alpha, in the current study we present two other reliability coefficients—omega hierarchical (ω_h_) and the IRT-based marginal reliability—which are useful when there are multiple items measuring a latent construct.

Statistics such as the omega hierarchical (ω_h_) (McDonald, 2013; Zinbarg et al., 2005) have been utilized as a more appropriate measure, especially when there are multiple items that capture a common underlying construct; in this case, the Science Self-Efficacy construct is formed using the undergraduates’ responses to the set of six items. The ω_h_ value is useful as it captures the structure of the test and the reliability of the general factor of the set of items; it is the ratio of the sum of correlations reproduced by the general factor to the sum of all correlations. Lastly, we also calculated an IRT-based reliability measure for unidimensional models (Thissen & Wainer, 2001).

Third, to assess the construct validity of the scale within the IRT framework, we fit a graded response model to the six polytomous items. IRT is a model-based measurement approach that views an individual’s probability of responding to an item as a result of both that individual’s latent trait (e.g., science self-efficacy) and the characteristics of the item (Embretson & Reise, 2000). Unlike classical test theory, which focuses on total scores, IRT concentrates on item-level analysis, making it ideal for examining the unique characteristics of individual items and how these characteristics might differ across various respondent subgroups, even when those groups have similar levels of the latent trait. This approach enables the identification of critical issues such as differential item functioning or item biases. All the analyses were conducted using the multidimensional item response theory, or mirt (Chalmers, 2012) package, in R software (R Core Team, 2022). To assess model fit, we used goodness-of-fit indices as before, such as the RMSEA, TLI, SRMSR, and the comparative fit index (CFI). The rule of thumb for these statistics is similar to interpreting the goodness of fit in CFA models (Schermelleh-Engel et al., 2003), that is, RMSEA  ≤ 0.05, SRMSR  <  0.05, and TLI and CFI  ≥  0.97 indicate a good fit.

We examined the measurement invariance and conducted a differential item functioning (DIF) analysis to examine whether students responded differently to the items based on their gender and race/ethnicity. If multiple items exhibit DIF, this may indicate the construct’s inability to utilize a common metric across different sub-groups. To assess DIF, we conducted Wald tests with Benjamini–Hochberg adjustment at the 0.05 alpha levels.

#### 2.3.3. Validity Based on Other Related Traits and Outcomes

Finally, as noted earlier, science self-efficacy is associated with many important outcomes in STEMM, including academic and psychosocial outcomes. We examined the relationship of the SSES with science identity and undergraduate degree completion (coded as 1 if graduated and 0 otherwise). In particular, we examined the validity of the IRT-scored SSES to assess whether undergraduate science self-efficacy measured in 2019 predicted completion of a bachelor’s degree by 2022.

To assess the predictive validity of the IRT-scored SSES and graduation, our sample size dropped to 7903 from 10,029 since we did not have institutional record data on all students. Among the students in the remaining sample, 4701 (59%) graduated and 3175 (40%) did not graduate by 2022. We examined the relationship between the SSES and those who did and did not graduate by first establishing measurement invariance using a CFA model for the unidimensional construct. To assess invariance, we fit three sequential models. Model 1 assumed the configural invariance, wherein the same factor structure is imposed on both groups (e.g., graduated or did not graduate). Model 2 assumed weak invariance, wherein the factor loadings are constrained to be equal across groups. Model 3 assumed strong invariance, that is, the factor loadings and intercepts are constrained to be equal across groups. To assess the goodness of fit of the models, we made use of the Likelihood Ratio Test, which compares the three nested models. Lastly, we examined the differences across those who graduated or did not graduate and conducted post hoc tests using Tukey’s HSD (Tukey’s Honest Significant Differences) test to assess the multiple pairwise comparisons between the means of the groups.

## 3. Results

### 3.1. Descriptives and Response Percentages

In Table 1, we present the demographic characteristics of the sample. The final analytic sample size was 10,029. Approximately 60% of the sample identified as women and 29% identified as men. Regarding racial and ethnic identity, 4% identified as American Indian or Alaska Native (AI/AN), 21% identified as Asian, 33% identified as Latine, 16% identified as Black and/or African American, 24% identified as White, and less than 1% students identified as Native Hawaiian or Pacific Islander (NHPI).

Students’ majors included three broad categories of biomedical majors—almost 51% were enrolled in biomedical sciences and biomedical-related engineering, 12% were enrolled in biomedical social or behavioral sciences, and 37% were non-biomedical majors. Examples of majors that comprise the biomedical natural sciences category included biology, biochemistry or biophysics, health professions, and few biomedical-related engineering majors; the biomedical social or behavioral sciences category included psychology and other biomedical-related social sciences majors; and non-biomedical majors included history, English, geography, and music.

Next, we examined the response percentages across the six items that form the SSES (Table 2). Across all six items we observed that a higher percentage of students responded to the middle categories, namely “somewhat confident” and “very confident.” For example, with respect to Item 3 (determine how to collect appropriate data), 6% of students responded that they were “not at all confident,” 38% students were “somewhat confident,” and 11% were “absolutely confident.” Regarding Item 6 (integrate results from multiple studies), about 8% of students responded that they were “not at all confident,” 36% were “somewhat confident,” 27% were “very confident,” and about 12% were “absolutely confident” that they could integrate results from multiple studies.

### 3.2. Construct Validity

#### 3.2.1. Dimensionality

The inter-item correlations varied from 0.50 (between Items 1 and 2) to 0.75 (between Items 5 and 6). These correlations reveal that the items are related to one another. Prior to fitting the factor analytic models, the overall KMO for the sample was 0.90. A higher value for the KMO statistic indicates that the data are better suited to conduct factor analysis. The EFA and CFA results for the two split halves of the samples are presented in Table 3.

The EFA resulted in one factor with factor loadings ranging from 0.65 for Item 1 to 0.84 for Item 3. The model fit statistics indicated RMSEA = 0.15, SRMSR = 0.03, and TLI = 0.94. Hence, model fit was found to be satisfactory. The CFA for the second sample also indicated good model fit with the following model fit statistics: RMSEA = 0.12, SRMSR = 0.03, and TLI = 0.95. The EFA and CFA via the cross-validation approach suggest a high construct validity with a single-factor structure for the SSES.

#### 3.2.2. Reliability Coefficients

The Cronbach’s alpha for the reliability coefficient was 0.91. In other words, 90% of the total or observed variance is considered as the true variance due to the differences in the latent trait, and the rest is due to measurement error. The omega hierarchical (ωh) reliability coefficient was 0.86, indicating that a larger proportion of the variance in SSES scores is due to a general factor. Lastly, the marginal reliability, an IRT-based reliability measure for unidimensional models, was 0.91, which means that on average, across the range of the latent trait, 90% of the observed variance is the true variance. Overall, these three reliability coefficients indicate that the SSES is a reliable scale that can be adopted by researchers.

#### 3.2.3. Item Response Theory Model: Model Parameters

Within the IRT framework, we fit a graded response model to the six polytomous items. Model fit was found to be adequate with RMSEA = 0.122 (95% CI [0.11, 0.13]), SRMSR = 0.036, TLI = 0.96, and CFI = 0.97. While the RMSEA might seem to be above the acceptable value of 0.05, the SRMSR goodness-of-fit statistic is important for evaluating model fit as it provides the average effect size of model misfit (Maydeu-Olivares, 2015), which is below the 0.05 cutoff and indicates good model fit along with the TLI and CFI statistics.

**Figure 1:**
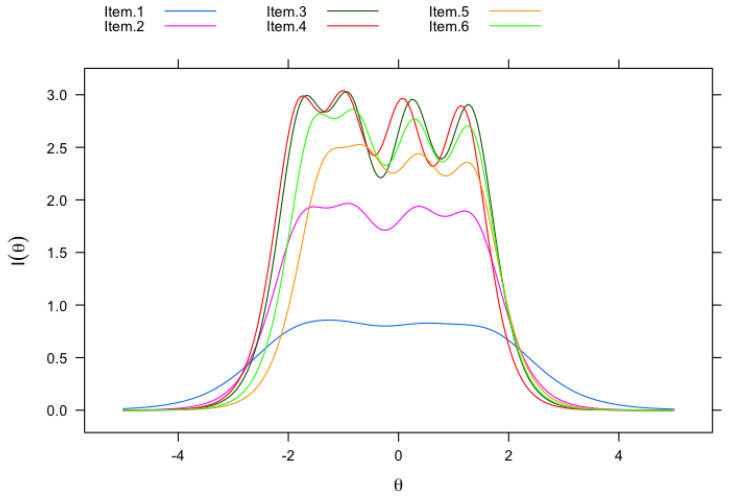
Item information curves (IICs) for the Science Self-Efficacy Scale.

The IRT parameters for the SSES are presented in Table 4. The latent trait captures students’ perceptions of how confident they are about their scientific skills. The slope, also known as the discrimination parameter, is a measure of how well an item differentiates responses of the undergraduates at different levels of the latent trait, that is, if the slope is steeper, the scale is better at differentiating among the various latent trait values. In Table 4, the slope parameter (*a*) for Item 1 (*use technical science skills*) is the smallest with a value of 1.69, and Items 3 (*determine how to collect appropriate data)* and 4 (*explain the results of a study*) have the largest value of 3.36. That is, Items 3 and 4 are more discriminating, with larger slopes, than Item 1. This means that Items 3 and 4 are more effective at distinguishing between respondents with similar latent trait levels, and therefore, they are more relevant to the construct than Item 1. This is also evident from Figure 1, which depicts the item information curves (IICs). The IIC for each of the items is a plot of the test information versus the latent trait, and it helps us understand the quality of the items across the latent trait, in other words, it shows how precisely each item measures the latent trait across the various levels of the SSES. The y-axis reflects the amount of information, indicating how well the items discriminate. Item 1 provides the least amount of information to the SSES, and Items 3 and 4 provide the most information to the SSES. This result echoes that Items 3 and 4 are more useful at differentiating respondents based on their trait levels than Item 1.

The threshold values or location parameters (b1 to b4 in Table 4) are the values of a latent trait that correspond to a 50% probability of responding at or above that location on an item. That is, b1 is the cutoff point of responding to category 1 vs. categories 2, 3, 4, or 5; b2 is the cutoff point of responding to categories 1 or 2 vs. categories 3, 4, or 5, and so on. For Item 1, the threshold parameters ranged from –1.86 to 1.63, that is, an undergraduate who has a science self-efficacy score of –1.86 has a 50–50 chance of selecting category 1; a student with a latent score of –0.97 has a 50–50 chance of selecting category 1 or 2 vs. categories 3, 4, or 5; and a student with a latent score of 1.63 has a 50–50 chance of selecting categories 1, 2, 3, 4 vs. category 5. The range of the item threshold parameter estimates appears reasonable across categories. We found that the undergraduates were not only responding to the highest or lowest categories but were endorsing all categories (see Table 2).

**Figure 2:**
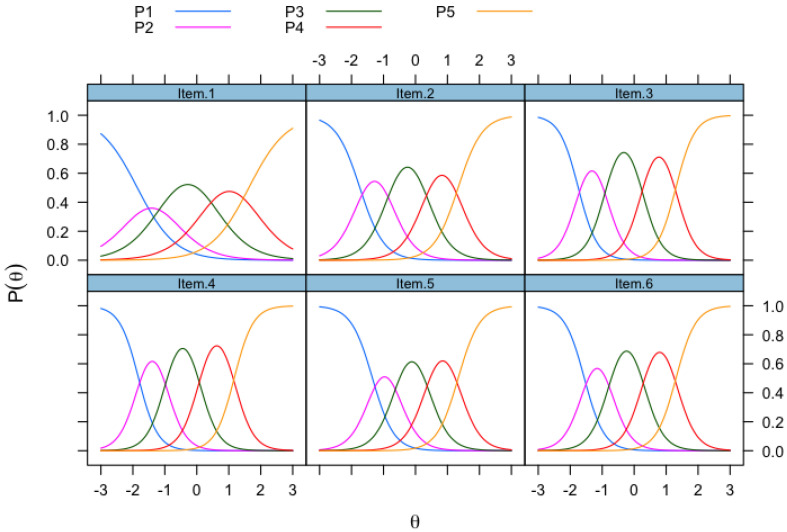
Category response curves (CRCs) for the Science Self-Efficacy Scale.

Figure 2 visually depicts the cut-offs between two curves, which is the threshold parameter (b) and the probability of endorsing a specific response category. The category response curves in Figure 2 help us understand the relationship between the items and the latent trait; that is, the curves represent the relationship between how students responded to the various response categories across the six items of the SSES. For example, for Item 1 (*use technical science skills*), the probability of endorsing category 4 (“very confident”) is approximately 42% in comparison to categories 3 or 5, which is only about 20%. Also, Item 1 has the least “peaked” curve for responses to category 2 (“a little confident”) in comparison to other categories (category 3 or 4). On the other hand, at the same latent trait of 1.0 for Item 4 *(explain the results of a study)*, for example, as shown in Figure 2, the probability of endorsing category 4 (“very confident”) is approximately 70% in comparison to category 5, which is much lower at about 21%. In other words, most of the students felt “very confident” in explaining the results of a study in comparison to “absolutely confident”.

#### 3.2.4. Item Response Theory Model: Differential Item Functioning

**Figure 3:**
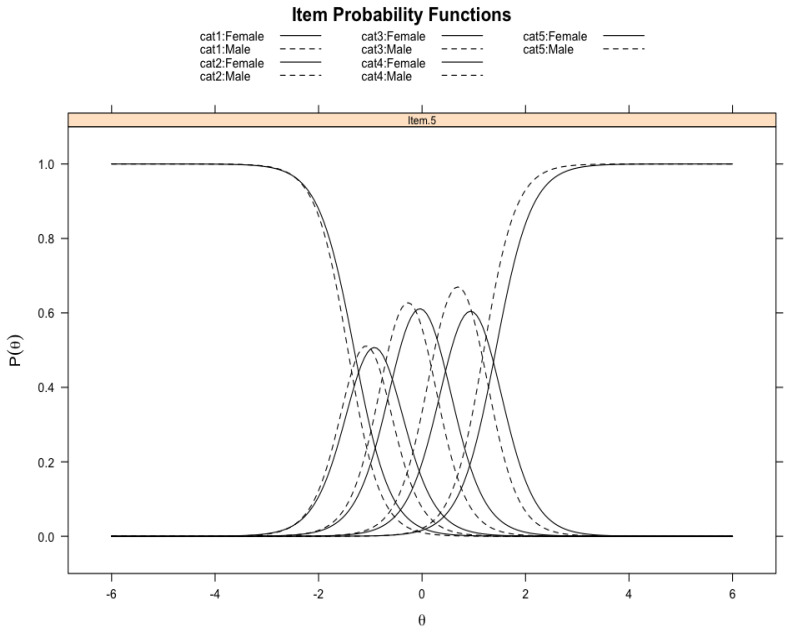
Item probability functions for women and men for Item 5 of the Science Self-Efficacy Scale.

Lastly, we examined DIF across gender and race/ethnicity. For all six items that make up the construct, we did not find any DIF across race/ethnicity^1^; however, we found DIF with respect to gender for Item 5 (see Table 5). In other words, we found that women and men were responding to or perceiving Item 5 (*use scientific literature to guide research*) differently. The item probability functions for item 5 (see Figure 3; solid lines correspond to women, and dashed lines correspond to men) indicate that women were responding differently to all the categories in comparison to men.

### 3.3. Validity Based on Related Traits and Outcomes: Science Identity and Graduation

We conducted analyses to examine the relationship between the SSES and other outcomes that are theoretically related to science self-efficacy. First, science identity and science self-efficacy were both scored using IRT and re-scaled to have a mean of 50 and standard deviation of 10. We found a correlation of 0.60 between the two continuous scales with respect to the IRT-scored science self-efficacy measure.

Next, to compare those who graduated versus those who did not, we compared the three nested models. In Table 6, we present the goodness of fit comparing Model 1 (configural invariance) to Model 2 (weak invariance), and then comparing Model 2 to Model 3 to assess strong invariance (last line of Table 6). In support of weak invariance (equal factor loadings), we found the *p*-value to be non-significant (*p* > 0.05, RMSEA = 0.003) and a smaller BIC for Model 2 compared to Model 1. Furthermore, when comparing the BIC values for Model 3 (BIC = 113,973) to those for Model 2 (BIC = 113,988), we found evidence for strong invariance and an RMSEA of 0.03 (which is considered a good fit). The results suggest invariance among the groups of students who graduated and did not graduate, allowing us to compare the latent means across these groups. The one-way ANOVA results found a statistically significant difference across undergraduates who graduated (*F* (1,7874) = 44.65, *p* < 0.001). Those who graduated had 1.46 points higher science self-efficacy scores compared to students who did not graduate with a baccalaureate degree.

## 4. Discussion

There is a dire need to support access and retention to STEMM training in order to keep up with national scientific workforce needs. To achieve these national workforce participation goals, it is vital to support undergraduate students by developing and boosting their perceptions of competence and confidence in science. To cultivate this capacity and build science career pathways leading to a diverse scientific workforce in all areas of biomedical research, we need valid and reliable measures that can help assess students’ confidence in their ability to conduct research and develop scientific skills. The SSES measures individuals’ beliefs about their self-competence in scientific research. SSES validation across a national diverse sample of students in the United States supports scale adoption across the larger U.S. population.

The comprehensive validation of the SSES and study of the item properties using item response theory are important for two main reasons. First, when considering the impact of self-efficacy, especially for underrepresented and disadvantaged groups in science, the literature suggests that self-efficacy is an outcome in and of itself that can provide support to students interested in STEMM through interventions/coursework (Carpi et al., 2017) as well as a driver or mediator of future outcomes, such as increasing aspirations for a STEMM career or persisting in one (Amelink et al., 2015). Second, one of the goals of the 10-year NIH BUILD initiative was to collect data across U.S. higher education institutions germane to developing measures to assess the long-term effects of key components of scientific training on undergraduates’ interest to pursue a career in biomedical research. Toward this end, the BUILD data sets will be available to researchers via the Inter-University Consortium for Political and Social Research (ICPSR) data repository in the future, and validated scales such as the SSES will be useful to researchers who want to adopt these scales in their research study.

We examined the psychometrics properties of the SSES with nearly 10,000 undergraduates from 11 higher education institutions across the United States. The results suggest that the science self-efficacy construct, composed of six items on an ordinal scale, is unidimensional, which was revealed using a cross-validation approach employing both exploratory and confirmatory factor analyses. The construct has strong reliability, which was examined via both omega hierarchical (ω_h_) and the IRT-based marginal reliability coefficients and good item-specific characteristics; that is, item discrimination and threshold parameters support the use of the construct composed of the six items across a wider pool of students. Together, these results lend strong support to construct validity. Additionally, we found that the items are invariant across race/ethnicity and that only one item, Item 5 (*use scientific literature to guide research*), indicated differential item functioning across women and me; thus, we recommend the adoption of the overall construct by STEMM researchers. It is important to know whether women and men are interpreting the items differently due to the way they perceive a given item or due to how the item is described.

Next, we examined validity based on related outcomes. We examined academic outcomes and psychosocial constructs that are theorized to be related to science self-efficacy. We examined the association between the SSES and science identity as well as between the SSES and graduating from college vs. not graduating. We found a strong relationship between the SSES and science identity, which aligns with previous research.

We found invariance among those who graduated vs. those who did not using a CFA model for unidimensional construct. Once the invariance was established, we were able to compare the latent means of the SSES across these groups. Our findings indicate that students who graduated had higher science self-efficacy than those who did not. Considering the items in the SSES, an important next step is to examine the relationship between the SSES and major at the time of graduation. This will allow future researchers to align the scientific skills needed to be successful in other majors (e.g., biomedical natural science and engineering) with the current wording of the items. Future work could include the development of items and possibly expanding the scale for undergraduates to other biomedical or non-biomedical majors.

### 4.1. Limitations

There are a few limitations in our study. First, we conducted a DIF analysis for race/ethnicity across only five race/ethnicity groups, and due to a small sample size of NHPI students, we were unable to include this group in our DIF analysis. This is important since there is a need to oversample students who identify as NHPI or AI/AN, not only to provide support for these subgroups of students but also to ensure that the scales or constructs are functioning similarly across subgroups. Second, the lack of qualitative data to support the current work and the post hoc nature of assessing the psychometric properties of the scale make it difficult to gain a deep understanding of why we might see differential item functioning across some subgroups. For example, while we can advocate for DIF across women and men for Item 5, we cannot say with certainty why that might be the case.

### 4.2. Recommendations for STEMM Research and Future Work

Enhancing the reliability and validity of scales like the SSES is essential for ensuring that researchers can accurately interpret and apply these measures when assessing the impact of interventions on students from underrepresented groups (e.g., Byars-Winston et al., 2016; Estrada et al., 2011). In particular, in social science research, scales are developed and used to capture behaviors, attitudes, or experiences that cannot be captured in a single variable or item. Using multiple items to measure an underlying latent construct can also isolate and account for item-specific measurement error, thus contributing to more accurate research (Boateng et al., 2018). For example, our study reveals that the construct is invariant, and the items are perceived similarly across the various race/ethnicity subgroups, which supports its validity and reliability for adoption across the broader research community for all students irrespective of their race groups.

Future research can investigate the measurement invariance of the Science Self-Efficacy Scale across assessment points. Establishing the measurement invariance of the scale over time will allow us to track changes in the participating students’ science self-efficacy over multiple years, and before and after the intervention. Additionally, future research can examine the scale using graduate trainees and/or early-career trainees. For example, the original scale adopted by Chemers et al. (2011) had three more advanced items, such as “publish research in peer review outlets”, added specifically to capture graduate students’ views. Students responded to these additional items on a five-point scale that ranged from 1 (not at all confident) to 5 (absolutely confident). Future work can include these three additional items while administering the scale to undergraduates and graduates to gain insights about how confident undergraduates and graduates feel about their scientific skills.

## 5. Conclusions

A validated scale whose psychometric properties are well understood enables us to ensure that the items and the scale are perceived similarly across various subgroups within an institution’s student population (e.g., across gender identities, race and ethnicity, and first-generation status). It is vital to provide various stakeholders with accurate information regarding the use of a scale with specific subpopulations. This enables us to investigate systematic group differences, which in turn can inform specific interventions and/or programs designed to facilitate equal opportunity to participate fully in the STEMM workforce.

## Figures and Tables

**Table 1:** Demographics characteristics of the sample (*N* = 10,029).

Variables	*N* (%)
Gender	
Women	6030 (60.12)
Men	2935 (29.27)
Race/Ethnicity	
AIAN	388 (4.44)
Asian	1906 (21.79)
Black/AA	1389 (15.88)
Latine	2899 (33.14)
NHPI	78 (0.89)
White	2087 (23.86)
Major	
Biomed Sciences and Engineering	4281 (51.36)
Biomed Social/Behavioral Sciences	973 (11.67)
Non-Biomedical	3082 (36.97)

**Table 2:** Response percentages to the six items forming the Science Self-Efficacy Scale (*N* = 10,029).

		Response Percentages (%)				
Item Number	Item Description	Not at all Confident (1)	A little Confident (2)	Somewhat Confident (3)	Very Confident (4)	Absolutely Confident (5)
Item 1	Use technical science skills (use of tools, instruments, and/or techniques)	9.84	14.68	37.30	25.80	12.38
Item 2	Generate a research question	7.44	16.50	37.23	26.15	12.67
Item 3	Determine how to collect appropriate data	6.20	14.38	38.37	29.38	11.67
Item 4	Explain the results of a study	5.49	13.28	33.95	33.14	14.13
Item 5	Use scientific literature to guide research	12.15	17.76	33.35	24.99	11.75
Item 6	Integrate results from multiple studies	8.46	15.95	36.00	27.73	11.87

Note: Students were asked: “Indicate to what extent you are confident that you can complete the following tasks”.

**Table 3:** Factor loadings of the six items using exploratory factor analysis (EFA) and confirmatory factor analysis (CFA).

Items	Factor Loadings for the First Half of the Sample (EFA)	Factor Loadings for the Second Half of the Sample (CFA)
Item 1	0.65	0.66
Item 2	0.78	0.78
Item 3	0.84	0.84
Item 4	0.83	0.84
Item 5	0.81	0.80
Item 6	0.83	0.82

**Table 4:** Slope (a) and thresholds or location parameters (b) along with standard errors for Science Self-Efficacy Scale.

Items	a (SE)	b1 (SE)	b2 (SE)	b3 (SE)	b4 (SE)
Item 1	1.69 (0.03)	−1.86 (0.03)	−0.97 (0.02)	0.40 (0.02)	1.63 (0.03)
Item 2	2.66 (0.04)	−1.74 (0.03)	−0.82 (0.02)	0.32 (0.02)	1.33 (0.02)
Item 3	3.36 (0.06)	−1.75 (0.03)	−0.89 (0.02)	0.25 (0.01)	1.30 (0.02)
Item 4	3.36 (0.06)	−1.82 (0.03)	−0.96 (0.02)	0.08 (0.01)	1.17 (0.02)
Item 5	2.99 (0.05)	−1.34 (0.02)	−0.59 (0.02)	0.37 (0.01)	1.34 (0.02)
Item 6	3.23 (0.06)	−1.56 (0.02)	−0.76 (0.02)	0.28 (0.01)	1.31 (0.02)

**Table 5:** The DIF results across gender and race/ethnicity for the six items that form the Science Self-Efficacy Scale.

Items	Gender		Race/Ethnicity	
	Wald Statistic	Adjusted *p*-Values	Wald Statistic	Adjusted *p*-Values
Item 1	1.338	0.412	1.007	0.631
Item 2	3.816	0.152	0.017	0.898
Item 3	1.195	0.412	0.207	0.898
Item 4	0.017	0.927	1.346	0.631
Item 5	7.206	0.044	0.03	0.898
Item 6	0.008	0.927	1.411	0.631

**Table 6:** Goodness of fit statistics comparing the three models.

Model	df	BIC	RMSEA	ChiSq Difference	Pr (>ChiSq)
Model 1	18	114,028			
Model 2	23	113,988	0.00314	5.194	0.3927
Model 3	28	113,973	0.0355	29.792	1.621 × 10^−5^ ***

Note: *** *p* < 0.001.

## Data Availability

The DPC Data Sharing Agreement governs access to consortium-wide data. Access to the data used for this paper can be granted to researchers who complete and submit an application for a data request. All analyses using consortium-wide data must be pre-approved as spelled out in the Data Sharing Agreement. The code used in the analyses is available upon request to the corresponding author. In 2026, the EDS data will be publicly available by request at the University of Michigan’s ICPSR data repository.

## Note

1 With respect to race/ethnicity, we had five groups: Asian, AIAN, Black/African American, Latine, and White.
